# Non‐Thermal Food Processing for Plant Protein Allergenicity Reduction: A Systematic Review

**DOI:** 10.1002/fsn3.70430

**Published:** 2025-06-10

**Authors:** Susana Arteaga‐Marin, Dona Ayala‐Larrea, Anaberta Cardador‐Martínez, Carmen Tellez‐Perez, Juan Alberto Resendiz‐Vazquez, Maritza Alonzo‐Macias

**Affiliations:** ^1^ Tecnologico de Monterrey, School of Engineering and Sciences Santiago de Querétaro Qro Mexico; ^2^ Laboratory of Engineering Science for Environment LaSIE‐UMR‐CNRS 7356, Eco‐Intensification of Agro‐Industrial Eco‐Processes La Rochelle University La Rochelle France

**Keywords:** allergen modification, food allergy, immunoreactivity, non‐thermal processing, plant proteins

## Abstract

The global incidence of food allergies has increased in the last decade. Compared with thermal processing techniques, non‐thermal food processing poses an effective solution for reducing allergenicity while preserving the nutrient content of the food. This study aims to compile the latest advances in modifying plant allergens through non‐thermal technologies. The information presented provides useful references for the potential production of hypoallergenic foods. We searched for original articles and reviews in databases published in English from January 2019 to August 2024. The databases used were ProQuest Central, ASC Journals, SAGE Journals, EBSCO Academic Search Ultimate, ScienceDirect, Web of Science, Springer Link, LILACS, Scopus, Cambridge University Press, and WIPO. Targeted allergen reduction and general protein structural modification were considered eligible for inclusion. Articles that did not determine structural or allergenicity modifications were excluded. Thirty‐five original research articles and three patents were included for review. No risk of bias assessment was performed. Results were synthesized manually in tables highlighting the principal results and confidence intervals for quantitative results. Findings indicate that non‐thermal processing technologies have the potential to effectively reduce plant protein's immunoreactivity by more than 50% compared to untreated plants when adequate conditions are selected. Allergenicity reduction is most probably achieved through protein structural modifications. Therefore, evidence of changes in secondary and tertiary structures produced after non‐thermal processing could represent the potential to use these techniques for future research on producing hypoallergenic foods. However, there are limitations in this relation as the structural changes could still not be sufficient to destroy protein epitopes or generate new antigenic sites. Treatments should be optimized for maximum immunoreactivity reduction while maintaining the plant's nutritional value and organoleptic properties.

## Graphical abstract

Non‐thermal food processing techniques can reduce allergenicity while preserving nutritional and sensory qualities. This study reviews articles and patents highlighting that non‐thermal methods can reduce plant protein immunoreactivity by over 50% through structural modifications.

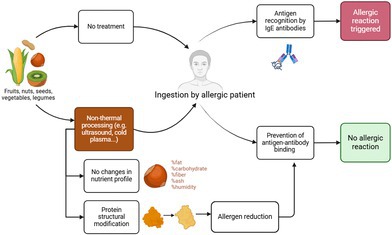

## 1 Introduction

The food industry is shifting as consumers increasingly reduce their consumption of animal‐derived foods in favor of plant‐based alternatives. Conventional farm practices are questioned regarding the environmental and ethical issues that meat production implies (Dong et al. 2024). Plant proteins, on the other hand, require less energy and water resources for their growth, and their consumption in the right quantities can often substitute the needed nutritional requirement for a healthy human diet at all ages (Dhiman et al. 2023; Goyal et al. 2024). Despite the positive health and environmental effects, plant‐based food consumption is often limited by the presence of food allergens that could trigger severe immunologic reactions. Almost all plant allergens are proteins belonging to the prolamin and cupin superfamilies (Costa et al. 2022). Protein allergens often resist heat, gastric digestion, and harsh pH conditions. Therefore, non‐thermal technologies offer a potential strategy for the structural modification of these allergens through physical treatments that can break down the structures that unite with antibodies (epitopes) and trigger an undesired immunological response (Narciso et al. 2024; Wang, He, et al. 2021). It is essential to study the application of non‐thermal technologies in the structural modification of plant proteins to assess the advances in producing nutritional hypoallergenic foods. Evidence of the effect of non‐thermal technologies on different food matrices and plants has been previously narratively reviewed. However, this is the first systematic review that focuses on plant allergen modification and aims to ensure reproducibility and reduce bias when presenting the final results compared to a narrative review.

This review evaluates the effects of non‐thermal technologies, including high‐pressure processing, cold plasma, ultrasound, electric fields, enzymatic digestion, and bacterial fermentation, on the secondary or tertiary structure modification of plant proteins and allergens compared to their corresponding untreated control proteins.

## 2 Methods

This systematic review was performed according to PRISMA guidelines (Page et al. 2021). The literature search was done between August 22nd–25th, 2024, and the databases used were: ProQuest Central, ASC Journals, SAGE Journals, EBSCO Academic Search Ultimate, ScienceDirect, Web of Science, Springer Link, LILACS, Scopus, Cambridge University Press, and World Intellectual Property Organization (WIPO). All the databases were searched through the BiblioXplora search engine (Tecnologico de Monterrey; https://biblioteca.tec.mx/). The early‐stage inclusion criteria established for the database search are original papers published in indexed scientific journals in the last 6 years (2024–2019) published in English. Reports focusing on plant food allergen reduction or allergy‐inducing plant protein structure modification through non‐thermal technologies will be included.

Following the criteria established, the search strategy (((allergen OR immunoreactivity OR allergenicity) AND (modification OR reduction)) AND non‐thermal AND technology) AND “PLANT PROTEIN” AND processing was used in the search query section in the advanced search for all databases applicable or in the simple search section for all databases that lacked an advanced search section. Changes in the search query were manually adapted if the search yielded no results. Web of Science used the search strategy with the operator ALL() at the beginning of the query to obtain results with the words present in any part of the document. LILACS search was modified to (allergen or immunoreactivity or allergenicity) AND (modification or reduction) AND (non‐thermal) AND (technology) AND (plant protein) AND (processing).

The results obtained in every database were limited to research articles published in English from January 2019 to August 2024, that were open or full access. Older relevant studies were not considered to guarantee the inclusion of studies that reflect the current state of non‐thermal processing for allergen and protein modification. A defined search period was delimited to ensure reproducibility. Extra limits “scientific journals,” “article,” “main article,” “complete text,” and “articles evaluated by experts” were added in ProQuest. EBSCO Academic Search Ultimate search included “academic journal” in the type of publication and “article,” “journal article,” and “research” in the type of document. The WIPO patent search included all offices, false stemming, faithful single family member, and false NPL. The patents published in 2024–2019 were manually selected from all the results. The limits established are by the inclusion criteria established for this review. The search strategy was peer‐reviewed by random assignation using PRISMA guidelines as a rubric.

**FIGURE 1:**
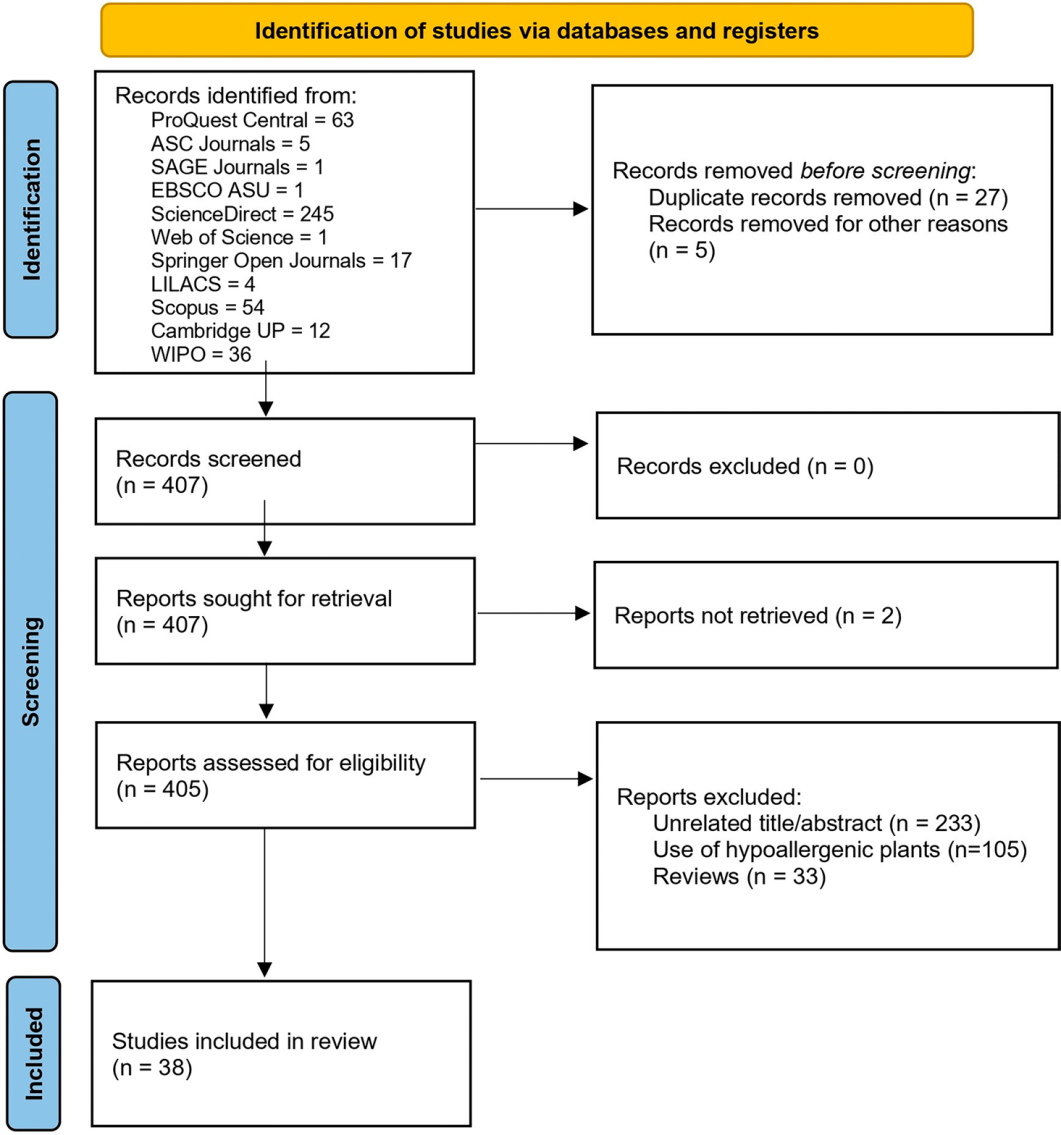
Flow diagram for literature search.

After database retrieval, screening first consisted of eliminating 27 duplicate studies and 5 records removed for being a type of document other than an article or review. The full text for two records could not be retrieved, leaving 405 records to be assessed for eligibility. Eligible studies should include components described in the PICO framework chosen for this review (Table 1). Figure 1 shows the flow diagram with the search methodology performed.

**TABLE 1:** PICO framework research process.

P (population)	I (intervention)	C (comparison)	O (outcome)
Plant protein	Non‐thermal food processing	Treated proteins vs. untreated proteins	Allergenicity or general protein structure modification

Studies that did not follow this PICO framework or with a different outcome of interest evaluated were considered ineligible. Titles and abstracts were first screened, and those that mentioned plant protein and protein modification with non‐thermal technologies were selected. Non‐thermal technologies included were ultrasound, microwaves, plasma treatment, fermentation, chemical conjugation, high‐pressure processing, enzymatic hydrolysis, extrusion, pulsed electric fields, and any variation of the technologies mentioned. Afterward, the whole text was screened for mentions of allergenicity. Records that did not address allergenicity or that focused on plant species with no known immunoreactive proteins (as identified by the World Health Organization) were deemed ineligible. Plant species for each article were searched as an allergen source in the WHO allergen nomenclature database (https://allergen.org/) and the search was limited to “food allergens” articles treating plants that yielded no results were excluded. The screening process was carried out manually by two authors without automation tools except for duplicate removal, which was performed with EndNote version 21.4. Two independent reviewers (S.A.‐M. and D.A.‐L.) screened titles and abstracts of each record retrieved. The two reviewers performed full‐text screening and collected data from every report according to the exclusion/inclusion criteria. Disagreements were resolved through discussion. A risk of bias assessment for non‐randomized in vitro experimental studies was performed using ChatGPT (GPT‐4o version); due to incompatibility with risk of bias standard tools, a customized framework was established to evaluate the risk of bias domains (Table 2).

**TABLE 2:** Customized risk of bias domains to evaluate the selected articles.

Domain	Assessment criteria
Selection bias	Was the selection of samples well described and appropriate?
Performance bias	Were treatment conditions clearly described and consistently applied?
Detection bias	Were detection methods for immunoreactivity or structure validated and consistently used?
Reporting bias	Were all outcomes reported transparently and without selective reporting?
Reproducibility	Were the experimental procedures described in enough detail to allow replication?

The outcomes evaluated in this review are the effect of non‐thermal processing on the protein secondary structure of allergy‐inducing plants. Not all non‐thermal treatments are intense enough to alter the primary structure; their effects are observed in changes of the secondary, tertiary, and quaternary structures. Most studies do not analyze the tertiary structure directly, as it requires highly purified protein samples, specialized expertise, and advanced equipment. Instead, changes in conformational epitopes are often inferred from alterations in the secondary structure (Zhou et al. 2021). The outcomes included could be either a percentage of change in immunoreactivity or changes in the distribution of the structural elements of any level of protein structures. Both domains were considered equally important and were compared to their respective untreated proteins, and effect sizes were interpreted as either statistically non‐significant or significant. Data were sought for the name of the non‐thermal technology used, treatment conditions, and the name of the target allergen if applicable. If the particular studied allergen were not mentioned, then it would be assumed that the authors worked with all allergenic proteins in the plant. Studies eligible for such synthesis must include immunodetection or protein secondary structure detection methodologies. Results were synthesized on tables displaying the primary outcomes and variables regarding the technology used. Studies were grouped by plant species for easier comparison. For this review, conducting meta‐analysis was impossible due to time constraints. Furthermore, no assessment of certainty was carried out.

## 3 Results

Non‐thermal technologies with proven results regarding allergenicity reduction include High Hydrostatic Pressure (Miguel Landim et al. 2023), cold plasma (Basak and Annapure 2022), fermentation, enzyme hydrolysis (Christensen et al. 2022), ultrasound (Jadhav et al. 2024), electric fields (Kheto et al. 2024; Rodrigues et al. 2020), etc. Studies selected that worked directly with any of these technologies on allergenicity reduction are summarized in Table 3. Allergenicity modification percentages were calculated based on the immunoreactivity of the control subjects. A study by Zhang et al. (2024) from the final selection was excluded as further analysis revealed that, although all inclusion requirements were present, the primary purpose was enhancing immunoreactivity for macrophage activation as a cancer treatment.

**TABLE 3:** Plant allergenic potential modification through non‐thermal processing.

Plant	Allergen	Technology	Conditions	Allergenicity modification	Confidence level	References
Sesame	N/A^a^	Low‐pressure plasma	25, 60, and 120 W	IgE binding capacity was not significantly reduced (5%)	95%	Dharini et al. (2023)
	2S albumins 7S globulins Oleosins	Ultrasound assisted extraction Microwave assisted extraction	450 W–20 min 500 W–20 min	All major allergen protein bands in SDS‐PAGE were degraded Alteration of the protein's primary structure	95%	Mathews et al. (2023)
Peanut	Ara h 1	Cold atmospheric pressure argon plasma jet	150 W for 5–50 times	IgG binding reactivity reduced by 38% using 5 times and 66% for 15 times	95%	Hsu et al. (2023)
	Ara h 1 Ara h 2	Cold plasma	52 kHz 32 kV	Allergenicity reduction of 65% for Ara h 1% and 66% for Ara h 2	95%	Venkataratnam et al. (2020)
	N/A^a^	Bacterial fermentation and autoclaving	44 h fermentation by *Bacillus natto*	Immunoreactivity reduced by 66% after autoclave alone Fermentation reduced IgE binding ability by 52%	95%	Pi et al. (2021)
Walnut	Walnut globulin	Polyphenol conjugation	Phenolic extracts from walnut pellicle (PEWP) via ultrasonication with concentrations from 1% to 20% pH 11 and 7	The alkaline system had significantly lower IgG‐binding capacity in walnut without PEWP 20% PEWP IgG‐binding capacity reached its lowest point	95%	Ma et al. (2024)
Cashew	Ana o 1 Ana o 2 Ana o 3	Instant controlled pressure drop	3.6–7 bar 43–120 s	IgE binding reduction of 67.2% with the most drastic treatment	95%	Vicente et al. (2020)
Pistachio	Pis v 1 Pis v 2 Pis v 3 Pis v 4	Instant controlled pressure drop	3.6–7 bar 43–120 s	IgE binding reduction of 75% with the most drastic treatment	95%	Vicente et al. (2020)
Tartary buckwheat	N/A^a^	Fermentation	24 h *Pediococcus pentosaceus*	IgE reactivity decreased by 82.8%	95%	Zhou et al. (2023)
Maize	N/A^a^	Enzymatic hydrolysis	Incubation of protein fractions with alcalase for 150 min at 30 min intervals	8 out of 35 peptides produced were regarded as probable allergens	95%	Sharma et al. (2022)
Kiwifruit	Act d 2	High intensity ultrasound	20 kHz 400 W 0–16 min	Highest time reduced Act d 2 content by 50% No significant changes observed in treatments shorter than 12 min	95%	Wang, Wang, et al. (2021)
		Microwave	75°C 0–5 min	Allergen content reduced by 80% for 5 min IgE binding capacity decreased the longer the treatments	95%	Wang, Zhang, et al. (2023)

^a^ The article does not specify the detection of a specific allergen but rather the detection of all allergenic proteins.

Most studies showed more than 50% decreased IgE or IgG binding ability (see Table 3) with the most intense treatment conditions, such as longer duration, higher pressure, or increased electrical current; except for low‐pressure plasma on sesame that had no statistically significant results between treatments (Dharini et al. 2023). All studies report a confidence interval of 95% for the statistical analysis of their results. However, two studies do not report a percentage of immunoreactivity reduction. Mathews et al. (2023) mention changes in the primary structure of sesame‐allergenic proteins but do not report a percentage of the reduction of IgE binding, which represents a reduction in the certainty of the results. Since Sharma et al. (2022) determined allergenicity through a bioinformatics tool for allergenicity prediction, results could be inaccurate if tested in vitro *or* in vivo.

**FIGURE 2:**
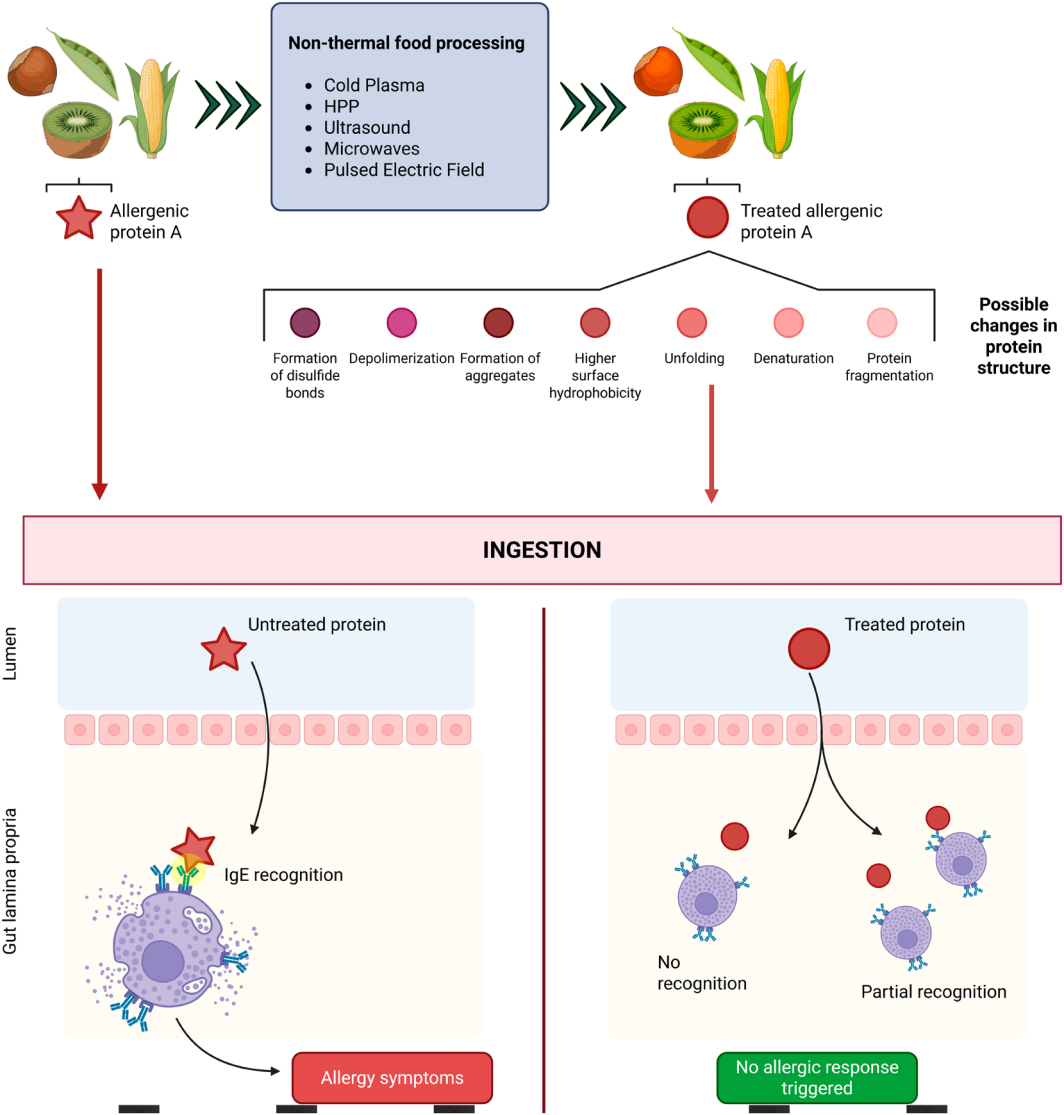
Theorized mechanism for immunoreactivity reduction via modification of conformational epitopes through non‐thermal food processing. IgE recognition of conformational epitopes in protein A contained in untreated peanuts (left) after digestion is carried out after antibody–antigen binding resulting in the release of inflammatory response mediators that trigger allergy symptoms in the patient. The same protein A can be found in treated peanuts but in its unfolded state (right) due to non‐thermal processing; the conformational changes avoid IgE recognition, therefore avoiding the immune response.

The exact mechanism by which non‐thermal technologies achieve a reduction in immunoreactivity remains unclear. However, it is theorized that physical treatments disrupt conformational or linear epitopes, avoiding the union antigen–antibody once the patient consumes the food (Figure 2). Spatially adjacent amino acids create conformational epitopes, meanwhile linear epitopes are continuous amino acids in the polypeptide chain (Chizoba Ekezie et al. 2018; Huang et al. 2024). Some authors attribute the reduction of allergenicity to changes in the tertiary, secondary, and even primary structure of proteins (Venkataratnam et al. 2020).

Changes in epitopes can be achieved through various mechanisms depending on the non‐thermal technology employed. Cold plasma changes in protein and allergen structure are mainly attributed to the action of plasma reactive species which cause the formation of disulfide bonds, depolymerization, fragmentation, masking of IgG binding epitopes, formation of insoluble aggregates, amino acid alteration, cross‐linking, and precipitation (Dharini et al. 2023; Hsu et al. 2023; Venkataratnam et al. 2020). The effect of High‐Pressure Homogenization on proteins includes an increase in surface hydrophobicity, unfolding of the spatial structure, rearrangement, aggregation, new disulfide bonds, and damage to the folded structure due to turbulence and erosion (Yan et al. 2024). Similarly, electric fields change protein structure and aggregation patterns (Rodrigues et al. 2020). Microwave processing can reduce immunoreactivity of plant proteins by reducing solubility (low free S‐H content), denaturation and aggregation of proteins (Wang, Zhang, et al. 2023). Ultrasound is associated with an increasing collision frequency and cavitation effects that promote protein aggregation, breakdown of hydrogen bonds, and rupture of peptide chains (Wang, Wang, et al. 2021; Yan et al. 2024). Furthermore, irradiation treatments may induce protein fragmentation, aggregation, cross‐linking, and amino acid modification (Wang, He, et al. 2021).

Therefore, studies dealing with structural changes induced by non‐thermal processing offer valuable insight into the potential that these technologies offer for producing hypoallergenic foods. Table 4 shows that most technologies can impact the proportion of secondary structures compared to untreated proteins. Changes in protein structure such as rearrangement of disulfide bonds, alterations in hydrophobic groups, and changes in the content of carbonyl groups can be measured through techniques like FT‐IR and SDS‐PAGE and indirectly measured through determination of protein solubility, hydrophobicity, and foaming capacity (Shen et al. 2023; Shrestha et al. 2023). Differences in the protein structure measurement methods among the articles may impact the comparability of the results due to differences in method sensitivity. Measurement of changes in protein structure should be made using two different methods for better validation of results. There is a lack of standardized protocols to measure changes in the secondary structure of proteins for allergenicity reduction; therefore, a direct comparison may be misleading.

**TABLE 4:** Conformational changes on plant proteins achieved by non‐thermal processing.

Plant	Technology used	Conditions	Results	References
Chickpea	Low frequency high intensity ultrasound	150 W for 30 min	Increase of protein solubility and surface hydrophobicity	(Bi et al. 2022)
	High‐pressure homogenization	0–150 MPa 1–3 cycles	Increase in protein solubility, foaming and emulsifying	Ma et al. (2023)
	Ultrasound‐assisted extraction	50% and 100% amplitude 5–25 min 200 W	Increased solubility Protein unfolding Changes in secondary structure	Sudarsanan et al. (2023)
	Atmospheric pressure plasma jets	0–50 s 40 mm between sample and nozzle	Increased solubility except for prolonged treatments that resulted in decreased solubility Higher abundance of β‐sheets Reduced α‐helix content	Wang, Zhou, et al. (2023)
	pH shifting and cold plasma	pH 12 30 s	Increased solubility Higher abundance of β‐sheets and reduced α‐helix content	Wang, Zhou, et al. (2024)
Pea	Cold atmospheric plasma	Use of plasma reactive species O_3_ and OH	Increasing abundance of β‐sheets Decrease of α‐helix content	Bu et al. (2022)
	High‐pressure homogenization	30/50 MPa Three cycles	Breaking of non‐covalent bonds for 30 MPa Breaking of covalent bonds for 50 MPa Increased solubility	Luo et al. (2022)
	High‐intensity ultrasound	100–400 W for 5 min pH 7.0	Decreased α‐helix. Increased β‐sheet and β‐aggregates. Unfolded proteins. Higher solubility and surface hydrophobicity	Gao et al. (2022)
	High‐intensity ultrasound and pH‐shifting	20 min, 20 kHz, 40%, ~36 W/cm^2^ pH 12 for 30 s then neutralized to pH 7	Moderate unfolding. Improved solubility and emulsifying capacity Protein unfolding Exposure of hydrophobic groups	Ma et al. (2022)
	High‐pressure homogenization and ultrasound	HPH: 50 MPa—3 cycles US: 20 kHz 400 W 20 min	Increase in solubility Lower abundance of β‐sheets and increased α‐helix content	Yan et al. (2024)

	Ultrasound	20 kHz 57–60 W cm^2^	Exposure of hydrophobic groups Increased solubility Changes in proteins vicilin 7S were less sensitive	Sha and Xiong (2022)
Electric fields (PEF, DCEF, ACEF)	PEF: 5–15 kV/cm, DCEF: 3–4 kV/cm, ACEF: 5–25 V/cm	All EFs reduced α‐helix and increased β‐sheet. Affected sulfhydryl/disulfide balance and intermolecular interactions Enhanced emulsifying stability	Guo et al. (2024)
Soybean	High hydrostatic pressure	400 MPa	Increase of protein solubility and surface hydrophobicity Reduction of β‐sheet structure	Dehnad et al. (2023)
	High moisture extrusion	120°C 30–6000 bar	Changes in secondary and tertiary structure Increased abundance of β‐sheets No degradation of proteins observed	Högg et al. (2024)
	Dielectric‐barrier discharge plasma treatment	40 kV 12 kHz 1–4 min	Protein fragmentation Amino acid chain modification Cleavage of peptide bonds	Liu et al. (2021)
		25–35 kV 2–8 min	Surface hydrophobicity increased for 30 kV until 4 min before declining Only the highest time increased protein solubility Increase in β‐sheets and β‐turns. Decrease in α‐helix	Rout and Srivastav (2024)
		33.8 kV 1–5 min	Increase in β‐sheets and β‐turns. Decrease in α‐helix Disruption of peptide bonds Rearrangement of amino acids	Xu et al. (2022)
	Pulsed electric field and pH shifting	10 kV/cm pH 11	Protein unfolding Increased solubility Decrease in β‐sheets, and α‐helix	Wang, Wang, et al. (2023)
Lentil	High‐pressure homogenization	0–150 MPa	100 MPa unfolds proteins by disrupting disulfide bonds Treatments below 150 MPa showed decreased content of β‐sheet, α‐helix, and β‐turns Changes in protein solubility are dependent on the pH used	Parlak et al. (2024)
Almond	Ultrasound Microwave	200 W for 10–50 min 160 W for 45–225 s Hydrolysis with enzyme alcalase	Production of low molecular weight peptides with potential reduced allergenicity	Sari et al. (2024)
Sunflower	Microwave	70 W 350 W Defrost mode 36–84 s	Reduced polar amino acids Slight decrease in α‐helix, increase in β‐sheet Increased solubility and surface hydrophobicity Improved emulsion stability	Gultekin Subasi et al. (2022)
	Electromagnetic fields	70 W and 350 W Less than 100 s	Changes in protein tertiary structure Increase in α‐helix and decrease in β‐sheets contents	Subasi et al. (2023)

Most conformational changes reported are in secondary or tertiary structures since changes in the linear polypeptide chain would require more intense treatments that could affect the organoleptic properties and nutritional content.

Hypoallergenic food products are increasingly important within the food industry, as several recent patents have sought to reduce allergenicity in plant‐based food processing. High pressures (higher than 10 bars) for more than 10 s in combination with temperatures below 150°C have been used to reduce antinutritional factors and to improve the digestibility of protein‐rich seeds. Results showed that the IgE reactivity of treated seeds was almost entirely suppressed compared to raw seeds (Chesneau et al. 2018). Similarly, high‐pressure homogenization (HPH) has been patented as a pre‐processing step for preparing a plant‐based yogurt alternative. HPH with pressures from 0.1 to 200 MPa before bacterial fermentation has been shown to produce a non‐allergenic yogurt substitute made of a plant protein concentrate (Avi et al. 2023). Pressured extrusion with changes from 0.2 to 3 to 0.8 bar allowed the production of a texturized hemp meat alternative without potential allergenic components (Ellis 2023). However, it is essential to mention the limitations of the last two patents as the inventors did not include immunoreactivity assay results to support their claims.

**FIGURE 3:**
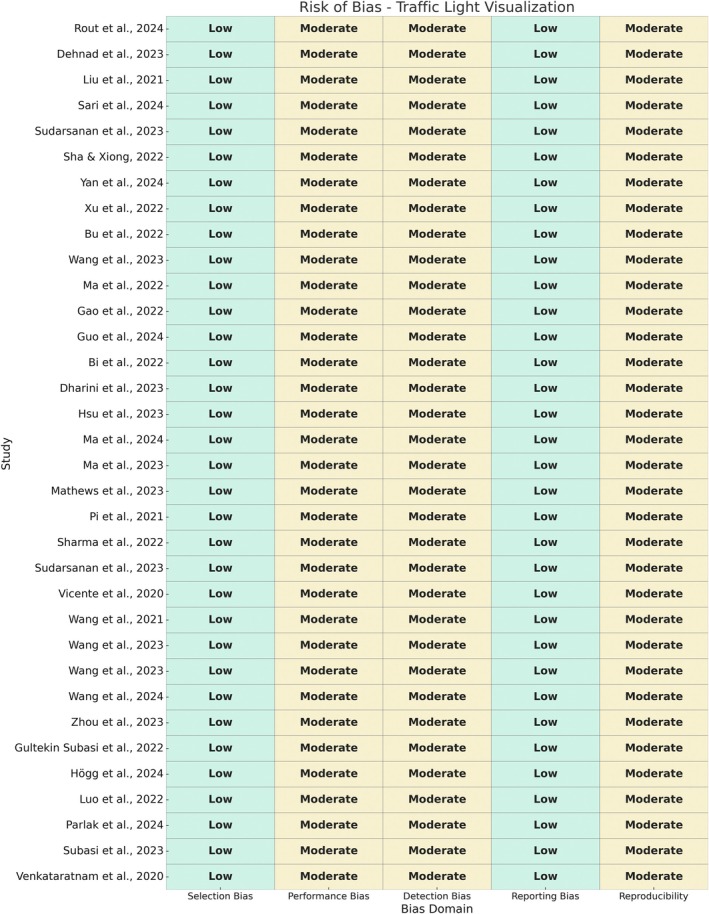
Summary of the results for the risk of bias assessment.

The risk of bias assessment resulted in a low to moderate bias level for all articles selected (Figure 3). A study quality assessment was not performed for this review.

## 4 Discussion

Plant‐based foods are novel alternatives to the traditional food supply that offer a more sustainable option while possessing unique nutritional and flavor profiles that meet human health needs. Despite the benefits of plant‐based foods, there are risk factors such as allergenicity that pose a challenge to incorporating plant proteins as meat alternatives completely into the human diet (Wang, Zhao, et al. 2024).

Food processing techniques are a key option to control food allergies. For example, food processing with heat may lead to reduced solubility of proteins, which in turn reduces immunoreactivity, as soluble allergens are more likely to trigger an immune response (Camus‐Ela et al. 2025). Even though thermal technologies have shown a potential to reduce the allergenicity of foods, non‐thermal technologies are preferred since they have less of a detrimental effect on nutritional value and food quality attributes than thermal technologies. The possible reason is that non‐thermal processing generally affects non‐covalent bonds, whilst thermal processing affects both covalent and non‐covalent bonds. This effect can be potentized by integrating more than two technologies in plant protein treatments (Wang et al. 2025). Furthermore, non‐thermal processing allows for retaining the maximum concentration of bioactive compounds and natural flavors (Allai et al. 2023; Liu et al. 2020). Non‐thermal technologies may also aid in binding bioactive compounds to mitigate the immunoreactivity of plant proteins. Xu et al. (2025) used ultrasound pretreatment for its cavitation effect on soybean to promote the binding efficiency of polyphenol punicalagin to soybean allergen protein 7S.

The compiled results show that non‐thermal food processing has the potential to reduce immunoreactivity by more than half of its normal value. Considering that food allergens are commonly heat‐resistant, the conditions with the ability to denature the allergenic proteins are normally the harshest. The number of studies that use these technologies for allergenicity reduction in plants is limited due to non‐thermal technologies being considered “emergent” technologies that are not so commonly used in the food industry. Therefore, it shows a potential area of research to improve global food safety. Since changes in immunoreactivity imply changes in protein structure, the analysis of studies that dealt only with structural changes was included as proof of the potential to produce hypoallergenic foods. Before measuring immunoreactivity, it is imperative that proteins be characterized first to observe any changes in disulfide or hydrogen bonds and even peptide bonds that could suppress epitopes. Even though the conformational changes achieved by non‐thermal processing depend on the technology used and the conditions, most studies achieve increased solubility and surface hydrophobicity, which is often sought in the elaboration of plant‐based foods. Additionally, there is a lack of patents for hypoallergenic foods with robust testing for immunoreactivity in vivo *and* in vitro.

Most studies in Table 1 were limited to in vitro immunoreactivity assays, so there is a limitation regarding how the allergenicity of treated food matrices manifests in patients. There is a need for in vivo allergenicity testing using allergic animal models for more accurate results that consider food digestion and the formation of novel epitopes due to changes in conformation. Furthermore, none of the studies included a sensory evaluation or estimation of organoleptic properties of the treated plants.

Due to time constraints, it was impossible to conduct a certainty assessment, which could have introduced a risk of error in the results. Although this assessment was not performed, the review nonetheless provides valuable insights into the potential production of hypoallergenic foods. Including a certainty assessment and meta‐analysis in future research could help with a more robust interpretation of results backed up with statistical analysis. The time was restricted from 2019 to August 2024, and the language was limited to English. Therefore, new studies or studies in different languages could have affected the robustness of the findings. Still, we are confident that the conclusion regarding the potential of non‐thermal processing in allergenicity reduction would not change.

## 5 Conclusion

Non‐thermal food processing technologies show potential for allergenicity reduction through protein structural modifications. These findings contribute to the rising need for obtaining safe and nutritious foods that can feed the entire population without risking the lives of sensitized people. Substitution of conventional food processing techniques with non‐thermal technologies ensures the conservation of nutrimental compounds and avoids drastic changes in rheological and sensory properties. Further studies on allergenicity reduction through non‐thermal technologies are advised to evaluate changes in nutritional content and physical properties, especially those with better results at the most intense treatment conditions. Furthermore, the technologies that were used exclusively to evaluate protein structural changes should be evaluated to determine if the changes observed are fit to modify conformational or linear epitopes. Moreover, research on hypoallergenic food products helps achieve global food security and prevent life‐threatening allergic reactions.

## Author Contributions

**Susana Arteaga‐Marin:** conceptualization (lead), data curation (lead), investigation (lead), methodology (lead), writing – original draft (lead). **Dona Ayala‐Larrea:** data curation (supporting), methodology (supporting), writing – original draft (supporting). **Anaberta Cardador‐Martínez:** resources (equal), supervision (equal), visualization (equal), writing – review and editing (equal). **Carmen Tellez‐Perez:** resources (equal), supervision (equal), visualization (equal), writing – review and editing (equal). **Juan Alberto Resendiz‐Vazquez:** visualization (equal), writing – review and editing (equal). **Maritza Alonzo‐Macias:** project administration (lead), resources (equal), supervision (equal), visualization (equal), writing – review and editing (equal).

## Conflicts of Interest

The authors declare no conflicts of interest.

## Data Availability

This systematic review was not registered, and the review protocol was not prepared. Other research data is not publicly available.
